# Infectious viruses from transfected SARS-CoV-2 genomic RNA

**DOI:** 10.3389/fbioe.2023.1129111

**Published:** 2023-03-30

**Authors:** Elina Väisänen, Miao Jiang, Larissa Laine, Matti Waris, Ilkka Julkunen, Pamela Österlund

**Affiliations:** ^1^ Expert Microbiology Unit, Department of Health Security, Finnish Institute for Health and Welfare, Helsinki, Finland; ^2^ Infection and Immunity Unit, Institute of Biomedicine, University of Turku, Turku, Finland

**Keywords:** SARS-CoV-2, infectivity, genomic RNA, biosafety, transfection

## Abstract

SARS-CoV-2 emerged at the end of 2019, and like other novel pathogens causing severe symptoms, WHO recommended heightened biosafety measures for laboratories working with the virus. The positive-stranded genomic RNA of coronaviruses has been known to be infectious since the 1970s, and overall, all experiments with the possibility of SARS-CoV-2 propagation are carried out in higher containment level laboratories. However, as SARS-CoV-2 RNA has been routinely handled in BSL-2 laboratories, the question of the true nature of RNA infectiousness has risen along with discussion of appropriate biosafety measures. Here, we studied the ability of native SARS-CoV-2 genomic RNA to produce infectious viruses when transfected into permissive cells and discussed the biosafety control measures related to these assays. In transfection assays large quantities of genomic vRNA of SARS-CoV-2 was required for a successful production of infectious viruses. However, the quantity of vRNA alone was not the only factor, and especially when the transfected RNA was derived from infected cells, even small amounts of genomic vRNA was enough for an infection. Virus replication was found to start rapidly after transfection, and infectious viruses were detected in the cell culture media at 24 h post-transfection. In addition, silica membrane-based kits were shown to be as good as traditional TRI-reagent based methods in extracting high-quality, 30 kb-long genomic vRNA. Taken together, our data indicates that all transfection experiments with samples containing genomic SARS-CoV-2 RNA should be categorized as a propagative work and the work should be conducted only in a higher containment BSL-3 laboratory.

## Introduction

Severe acute respiratory syndrome coronavirus 2 (SARS-CoV-2) emerged at the end of 2019 in China (Zhu et al., 2020) and then rapidly spread throughout the world causing one of the most severe pandemics in human history. Infection with SARS-CoV-2 causes a disease (COVID-19) with symptoms varying from mild and moderate common cold-like symptoms to a severe respiratory tract infection which with other complications may lead to death (World Health Organization, 2022). Similar to other novel pathogens causing severe symptoms, the World Health Organization (WHO) recommended heightened biosafety control measures for laboratories working with SARS-CoV-2 (World Health Organization, 2020), and in Finland, SARS-CoV-2 was categorized as a risk group 3 pathogen in 2020 (Ministry of Social Affairs and Health, 2020). However, already in January 2020 WHO stated that diagnostic molecular testing of patient samples can be handled similarly to samples from suspected human influenza cases, meaning that BSL-2 facilities can be used (World Health Organization, 2020). Currently, the guidelines have remained the same: the WHO Laboratory Biosafety Guidance Related to Coronavirus Disease states that all SARS-CoV-2 propagative work should be conducted exclusively in the higher containment level laboratory, whereas the non-propagative diagnostic work, including nucleic acid testing and sequencing, can be performed in a BSL-2 laboratory (World Health Organization, 2021).

SARS-CoV-2 has a long, nearly 30 kb, non-segmented, positive-stranded RNA genome (Zhu et al., 2020). This characteristic is shared with all coronaviruses, and such RNA genomes are among the largest ones known even when considering the viruses with segmented RNA genomes (Masters, 2006; Campillo-Balderas et al., 2015). The positive-stranded RNA genomes with 5’ cap structures and poly(A) tails can function directly as mRNA leading to viral protein synthesis and subsequently to viral RNA replication, in other words, the plain genomic vRNA can be infectious. For coronaviruses, the infectivity of the genomic vRNA was already demonstrated in the 1970s. The genomic vRNAs of the avian coronavirus infectious bronchitis virus (IBV), and mouse hepatitis virus (MHV) strain JHM, were shown to produce infectious viruses when transfected into permissive cells (Lomniczi, 1977; Wege et al., 1978).

More recently, the studies utilizing transfection of genomic coronavirus RNA have been focusing on reverse genetics, generating infectious cDNA clones of genetically manipulated coronaviruses, and secondly for analyzing the role of genomic vRNA on innate immunity (Yount et al., 2003; Almazán et al., 2006; Scobey et al., 2013; Thi Nhu Thao et al., 2020; Xie et al., 2020; Kouwaki et al., 2021; Fahnøe et al., 2022). One of the main drivers for reverse genetics has been the emergence of a highly pathogenic human SARS-CoV in 2002 (Tsang et al., 2003), MERS-CoV in 2012 (Zaki et al., 2012) and most recently SARS-CoV-2, which has created an urgent need for experimental systems for studying viral pathogenesis and immune responses as well as testing for candidate antivirals and therapeutics. In the studies creating cDNA clones, fragments covering the entire coronavirus genome are assembled into a full-length genomic cDNA, which is then transcribed *in vitro* into RNA. Finally, the RNA is transfected into permissive cells for viral rescue. The *in-vitro* transcription easily yields micrograms of genomic vRNA from the cDNA template, and such a large amount of vRNA has helped in the efficient rescue of viruses from transfected vRNA. On the other hand, in innate immunity studies, rescuing infectious viruses is not the main goal. As compared to the infectious cDNA studies, analysis of innate immunity requires less vRNA in the transfections, incubation times are often shorter, and the production of infectious viruses is usually not analyzed. Therefore, it is not known whether infectious viruses are produced in such studies. Overall, studies which include both the transfection of native or smaller quantities of viral RNA and the evaluation of the virus production, have been rare.

Here, we studied the ability of native SARS-CoV-2 genomic RNA to produce infectious viruses after transfection into permissive cells. As a model, we used a SARS-CoV-2 beta variant (Fin32 strain) RNA transfected into VeroE6-TMPRSS2-H10 cell line (VE6-T2). This variant is known to replicate efficiently in VeroE6 and VE6-T2 cells, and produces a clearly detectable cytopathic effect (CPE) during infection. To define the vRNA quantity needed for rescuing infectious virus from the transfection, we concentrated Fin32 viruses by ultracentrifugation, extracted the vRNA with TRIsure reagent, and measured the vRNA concentration. We also studied the timeline for infectious virus production from transfected cells. Finally, we investigated the ability of a silica-membrane based kit to extract 30 kb-long intact vRNA and its suitability for transfections as well as the capability of total cellular RNA from virus-infected cells to produce infectious viruses when transfected into VE6-T2 cells. Based on the obtained results the biosafety aspects related to the control measures when handling the genomic vRNA of coronaviruses are discussed.

## Material and methods

### Cells

VeroE6-TMPRSS2-H10 (VE6-T2) (Rusanen et al., 2021) cells were maintained in Eagle’s minimum essential medium supplemented with 60 μg/mL penicillin, 100 μg/mL streptomycin, 2 mM L-glutamine, 20 mM HEPES, and 10% fetal bovine serum (Sigma Aldrich). For transfection experiments, VE6-T2 cells were seeded into 24-well plates 24 h prior to transfection at a density of 1 × 10^5^ cells per well. For endpoint dilution assays, VE6-T2 cells were seeded into 96-well plates at a density of 3.75 × 10^4^ cells per well.

### Virus

SARS-CoV-2 strain hCoV-19/Finland/THL-202101018/2021 (Fin32, a beta variant (B.1.351), EPI_ISL_3471851, ON532063), was isolated and propagated in VE6-T2 cells as previously described (Laine et al., 2022)*.* The stock virus from passage 3 was used in the experiments. All experiments with infectious SARS-CoV-2 virus as well as all transfection experiments were carried out strictly under biosafety level 3 laboratory conditions at the Finnish Institute for Health and Welfare (THL), Finland.

### Viral RNA preparations

To obtain a high concentration of genomic vRNA, the Fin32 passage 3 virus stock was concentrated by sedimentation through a 20% sucrose cushion by ultracentrifugation at 26,000 rpm for 90 min in an SW32 rotor (Beckman Coulter). The pelleted viruses were either resuspended into PBS and lysed with TRIsure reagent (Meridian Bioscience) or directly lysed into RLT buffer (Qiagen) containing 1% β-mercaptoethanol. Genomic vRNA was extracted according to manufacturer’s instructions for TRIsure or RNeasy Mini kit, respectively.

**FIGURE 1:**
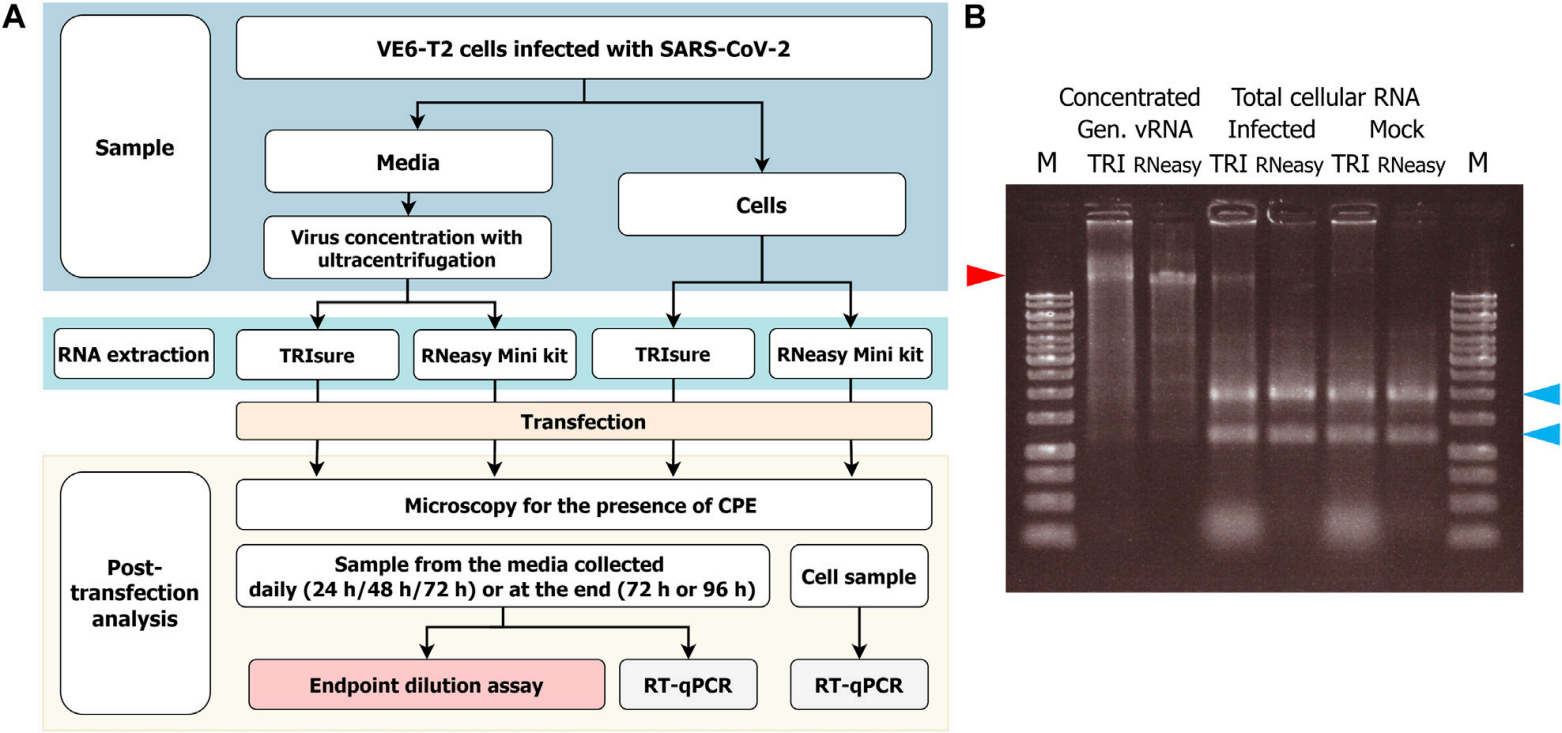
**(A)** Flowchart of the RNA transfection experiments and infectivity analysis. VeroE6-TMPRSS2 (VE6-T2) cells were infected with a low MOI of SARS-CoV-2 and vRNA was harvested either from the culture media or from infected cells. After RNA transfection to VE6-T2 cells, cytopathic effect (CPE) as an indication of infection was monitored, and media and cell samples were collected during 3–4 days of incubation. The infectious viruses in the media samples were titrated with cultivation in VE6-T2 cells in an endpoint dilution assay, or vRNA was quantified from the media or cell samples with RT-qPCR with SARS-CoV-2 E gene specific assay (negative control wells). **(B)** Gel analysis of the RNA on a native agarose gel. RNA samples (1 µg/well) were run on a 0.8% native agarose gel. RNA samples in the native state can form secondary structures that may run faster or differently than linear DNA ladder. Therefore, the size of vRNA or rRNA may appear lower than DNA ladder in the gel. M, GeneRuler 1 kb DNA ladder (Thermo Fisher; the highest band is 10 kb); TRI, RNA extracted with TRIsure reagent; RNeasy, RNA extracted with RNeasy Mini kit. The red arrow indicates the genomic vRNA of SARS-CoV-2, and blue arrows indicate 28S and 18S rRNA, respectively.

The experimental set up has been described in Figure 1A.

For transfection experiments, the total cellular RNA from VE6-T2 cells infected with Fin32 was collected at 24 h or 42 h post-infection and RNA was extracted either with TRIsure reagent or RNeasy Mini kit with DNase (Qiagen) treatment according to manufacturer’s instructions.

All RNA concentrations were measured with NanoDrop 1000 Spectrophotometer (Thermo Fisher Scientific).

### Gel electrophoresis

The extracted RNA batches were analyzed on native agarose gels. Briefly, RNA (1 µg/well) was mixed with RNA loading dye (in-house, 50% glycerol, 0.1 mM EDTA, with bromphenol blue and xylene cyanol FF dyes) and run on a 0.8% (w/v, SeaKem LE agarose, Cambrex) native agarose gel in TBE buffer (0.1 M boric acid, 0.1 M Tris, 2.5 mM EDTA, pH 8.3) with 90 V for 1 h. The gel was post-stained with 1 × GelRed Nucleic Acid Gel Stain (Biotium) in TBE buffer for 1 h. The image was acquired with GeneFlash device (Syngene Bio Imaging).

### Transfections

VE6-T2 cells were transfected using the following protocol: Lipofectamine 3000 (LF3000) (1–1.5 µL per well, Invitrogen) was diluted into Opti-MEM media (Gibco), and after 5 min incubation at RT, the diluted LF3000 was mixed with the RNA (200 ng of RNA per well or as indicated in the figures). The mixture was incubated for 20 min to allow RNA-lipid complexes to form, and then applied dropwise on the cells. Cells were incubated at + 37°C with 5% CO_2_ for 72 or 96 h. The formation of cytopathic effect as an indication of infection was monitored daily. Samples were collected either daily (from media) or at the end of the experiment (total cellular RNA and media). In addition to mock cells, negative controls included cells transfected with LF3000 without RNA, and cells onto which plain RNA was applied without any transfection reagent.

Transfection experiments were repeated 2–10 times for different RNA preparations with multiple replicates, except that the 500 ng vRNA experiment was done only once. The mean value of replicates in an individual experiment was 8.0 (median 10; min 2; max 11).

### Endpoint dilution assay

The presence of infectious viruses in the media post-transfection was analyzed with an endpoint dilution assay as previously described (Laine et al., 2022). Media collected from transfected cells showing a clear cytopathic effect (CPE) as well as media from all daily follow-up samples were analyzed with serial dilutions from 1:100 dilution onwards with eight replicates for each dilution. Transfected or control wells with no CPE were analyzed from 1:10 dilution with eight replicates. The CPE was observed at day five post infection, and the virus titer as TCID_50_ per ml was calculated using the Spearman-Kärber method.

### RT-qPCR

For RT-qPCR analysis of post-transfection samples, RNA from cell culture supernatants and total cellular RNA was extracted with the RNeasy Mini kit according to manufacturer’s instructions. For genomic vRNA and post-transfection RNA samples the quantity of SARS-CoV-2 RNA in the sample was analyzed with SARS-CoV-2 E or RdRp gene RT-qPCR as previously described (Corman et al., 2020; Laine et al., 2022). Briefly, 5 µL of genomic vRNA or a dilution of vRNA, 500 ng of total cellular RNA, or 5 µL of cell culture supernatant extracted RNA was reverse transcribed (RT) to cDNA. Then 5 μL of the cDNA was amplified with SARS-CoV-2 E or RdRp gene specific qPCR along with a plasmid standard curve of known concentrations (10^1^–10^7^ copies per µL). The Ct-value of the sample was compared to the Ct-values of the standard curve for SARS-CoV-2 E or RdRp gene quantity extrapolation, respectively, and from this the vRNA quantity in the initial sample was calculated. The SARS-CoV-2 sequences in the plasmids for E and RdRp genes used for the standard curves, respectively, are identical to hCoV-19/Finland/1/2020 sequence (GenBank MT020781). For genomic vRNA, both the E and RdRp gene quantities are considered to be equal to the viral genome quantity. With total cellular RNA from SARS-CoV-2 infected cells, the RdRp quantity indicates better the true amount of full-length viral genomes in the sample, whereas the E gene quantity includes both viral genomes and numerous subgenomic E transcripts.

### Digital PCR

QuantStudio 3D Digital PCR System (ThermoFisher Scientific) was used to confirm the SARS-CoV-2 RT-qPCR quantities. Reactions containing 1x Absolute Q 1-step Master Mix (ThermoFisher Scientific), 0.4 μM E gene forward and reverse primers, 0.2 μM E gene probe, and RNA template were loaded on 20K Chips v2. Chips were thermal cycled on ProFlex 2 Flat PCR System with the following protocol: 50°C for 10 min, 96°C for 10 min, 39 cycles of 60°C for 2 min and 98°C for 30 s, 60°C for 2 min, and 10°C until finished. Chips were read and analyzed using the QuantStudio 3D Digital PCR instrument and AnalysisSuite software.

### Statistics

GraphPad Prism 9.0 (GraphPad Software) was used for statistical analyses. One-way ANOVA with Tukey’s multiple comparisons test or unpaired *t*-test with Welch’s correction was used for the analysis.

## Results

### Virus concentration and genomic viral RNA extraction

To obtain native intact full size genomic vRNA, Fin32 virus was cultivated in VE6-T2 cells with a low MOI for 2–3 days and concentrated by ultracentrifugation. After RNA extraction, a total of 35–80 µg of genomic vRNA per one ultracentrifugation round was obtained. This corresponds to −10–20 µg of genomic vRNA per 50 mL of original stock virus, i.e., media from an infection of one T175 bottle. The obtained RNA quantity did not differ between the two extraction methods (TRIsure reagent or RNeasy Mini Kit). RNA was aliquoted and the quality was examined with agarose gel electrophoresis (Figure 1B).

### Transfection with SARS-CoV-2 genomic RNA

**FIGURE 2:**
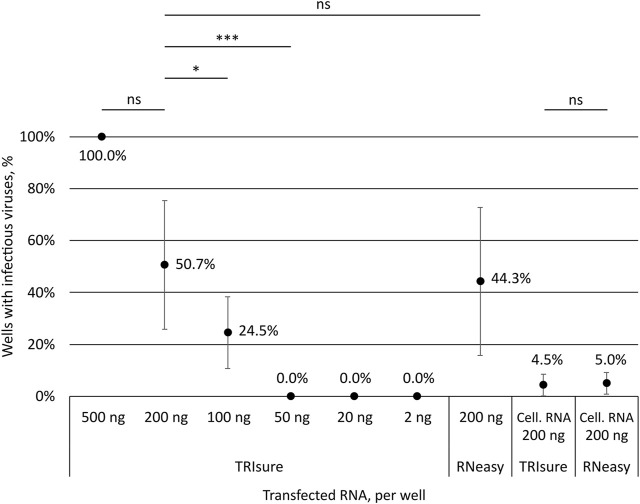
Correlation of the transfected RNA amount to the production of infectious viruses. Different amounts of SARS-CoV-2 vRNA (concentrated genomic vRNA or total cellular RNA from virus-infected cells) were transfected into VeroE6-TMPRSS2 cells and the formation of CPE by infectious viruses was monitored. The presence or lack of infectious viruses was confirmed by an endpoint dilution assay. The graph shows the percentage of wells with an infection after transfection (both CPE and TCID_50_ titer positive). The means and standard errors of the means have been calculated from the following number of experiments (the total number of replicates in parenthesis): 500 ng, 1 (3); 200 ng, 10 (63); 100 ng, 5 (39); 50 ng, 2 (15); 20 ng, 3 (30); 2 ng, 3 (30); 200 ng kit, 7 (58); Cell. RNA, TRIsure, 5 (46); Cell. RNA, RNeasy, 2 (20). Cell. RNA, total cellular RNA from virus-infected cells; TRIsure, RNA extracted with TRIsure reagent; RNeasy, RNA extracted with RNeasy Mini kit. *p* < 0.05 (*), *p* < 0.001 (***), not significant (ns).

To assess how much of genomic vRNA is needed to obtain infectious viruses from transfection, different amounts of TRIsure-extracted vRNA were transfected into VE6-T2 cells with LF3000. Transfected cells were followed up for 3–4 days and infectious viruses were determined by CPE and with an endpoint dilution assay from the supernatant samples. All wells transfected with 500 ng per well of vRNA produced infectious viruses followed by 50.7% of the wells transfected with 200 ng and 24.5% with 100 ng of vRNA per well (Figure 2). Although the infectivity correlated with the quantity of the transfected vRNA, i.e., more wells with infectious viruses were observed when more vRNA was transfected, there was a great variation between individual experiments. In some experiments all or nearly all wells transfected with 100 ng or 200 ng of vRNA produced infectious viruses whereas in other experiments only few or none of the wells had an infection. Nevertheless, no infectious viruses were observed in wells transfected with 50 ng or less of vRNA although a total of 75 wells were transfected either with 50 ng, 20 ng or 2 ng of vRNA (Figure 2).

In addition to the traditionally used TRI-reagent based RNA extraction methods, we wanted to analyze the ability of a silica-membrane based kit to extract −30 kb-long intact vRNA of coronaviruses. We extracted the genomic vRNA from concentrated viruses with the RNeasy Mini kit and transfected the vRNA (200 ng per well) with LF3000 into VE6-T2 cells. After extraction, a band corresponding to the size of genomic vRNA was seen on native agarose gel (Figure 1B), and furthermore, a similar rate of infectivity was observed from transfections with both RNeasy Mini kit-extracted and TRIsure-extracted vRNA (44.3% vs. 50.7% respectively, Figure 2.).

### Transfection with total cellular RNA from SARS-CoV-2 infected cells

When total cellular RNA from Fin32 infected cells (200 ng per well) was transfected into fresh VE6-T2 cells, infectious viruses were observed, although very rarely. On an average less than 5% of the transfected wells yielded infectious viruses, and the frequency was the same with both TRIsure and RNeasy Mini kit extracted total cellular RNA (Figure 2).

### Negative controls for transfections

No CPE was observed in negative control cells (mock), cells transfected with LF3000 reagent only or in cells onto which plain RNA without any transfection reagent was applied to. Also, the virus titration from the culture media remained negative for these samples.

### Quantification of vRNA and calculation of transfected vRNA copies per cell

The concentrated genomic vRNA was quantitated by SARS-CoV-2 E and RdRp gene-specific RT-qPCRs, and the quantities were confirmed by a digital PCR. As quantitated by RT-qPCR, 200 ng of SARS-CoV-2 vRNA contained 1 × 10^10^ to 5 × 10^10^ genome equivalents depending on the vRNA batch. As analyzed by a digital PCR the genome equivalents ranged between 9 × 10^9^ to 3 × 10^10^ (Table 1). This shows that the RT-qPCR quantification with both E and RdRp genomic regions was fairly accurate. Quantified genome equivalent values correspond to values that can be obtained through mathematical weight to molar calculations (for example, https://www.bioline.com/media/calculator/01_07.html) (Table 1).

**TABLE 1:** RNA mass vs. virus copies analyzed with different methods from different RNA samples. The variations are due to differences between RNA batches.

Type of RNA	Mass of RNA (ng)	E gene copies, RT-qPCR	RdRp gene copies, RT-qPCR	E gene copies, digital PCR	Weight to molar calculator*
Genomic vRNA	200	1 × 10^10^–5 × 10^10^	1 × 10^10^–2.5 × 10^10^	9 × 10^9^–3 × 10^10^	1.2 × 10^10^
Total cellular RNA from infected cells	200	1.7 × 10^9^–5 × 10^9#^	1.8 × 10^8^–5 × 10^8^	NA	NA

*https://www.bioline.com/media/calculator/01_07.html; #, includes both genomic vRNA and subgenomic E gene transcripts; NA, not analyzed.

For transfections, VE2-T2 cells were plated 24 h prior to transfection at a density of 1 × 10^5^ cells per well. Based on our transfection data and vRNA quantification, we estimated that transfection with >3 × 10^5^ genomic vRNA copies per cell yielded 100% infection. In addition, more than 4 × 10^4^ vRNA copies per cell were needed in the transfection to achieve any replication of infectious viruses when purified genomic vRNA from stock viruses was used in our experimental set up. However, when the SARS-CoV-2 genomic RNA was part of the total cellular RNA from infected cells that was used in the transfection, the required vRNA copy number for infectivity was up to 100-fold lower based on the RdRp gene quantification (Table 1).

The results of negative controls were further confirmed by SARS-CoV-2 E gene-specific RT-qPCR. Media samples from the mock cells and transfection reagent control cells were completely negative for SARS-CoV E gene. The unprotected, plain RNA was observed to be rapidly degraded from the media. Already at 1 h after applying 200 ng of genomic vRNA on the cells, no viral RNA was detected in the media or attached to the cells as analyzed by SARS-CoV-2 E gene RT-qPCR (n = 8 wells). In comparison, the highest reliably quantified amount of genomic vRNA (1:100 dilution of the vRNA, 2 ng) was giving a Ct value of 13, which corresponds to −1–5 x 10^8^ copies per 2 ng of vRNA or −1–5 x 10^10^ copies per 200 ng of vRNA. With total cellular RNA from infected cells, the E gene quantity was −3 × 10^9^ per 200 ng of total cellular RNA, however, this value includes both genomic vRNA and subgenomic E transcripts. When this RNA was applied into wells, a similar reduction in the quantity was observed: only traces (if any) of E gene was detected in the analyzed wells at 1 h (n = 8). The Ct value of the positive wells at 1 h in RT-qPCR was close to the detection limit of the assay (Ct values 38–40), which in supernatant samples corresponds to less than 5 E gene copies per qPCR reaction or ∼8 × 10^2^ copies per well. This shows that the cellular RNA was as sensitive to degradation as the pure genomic vRNA when applied onto the cells as naked RNA, without the protection from the lipid-based transfection reagent.

### Daily follow-up of transfected cells

**FIGURE 3:**
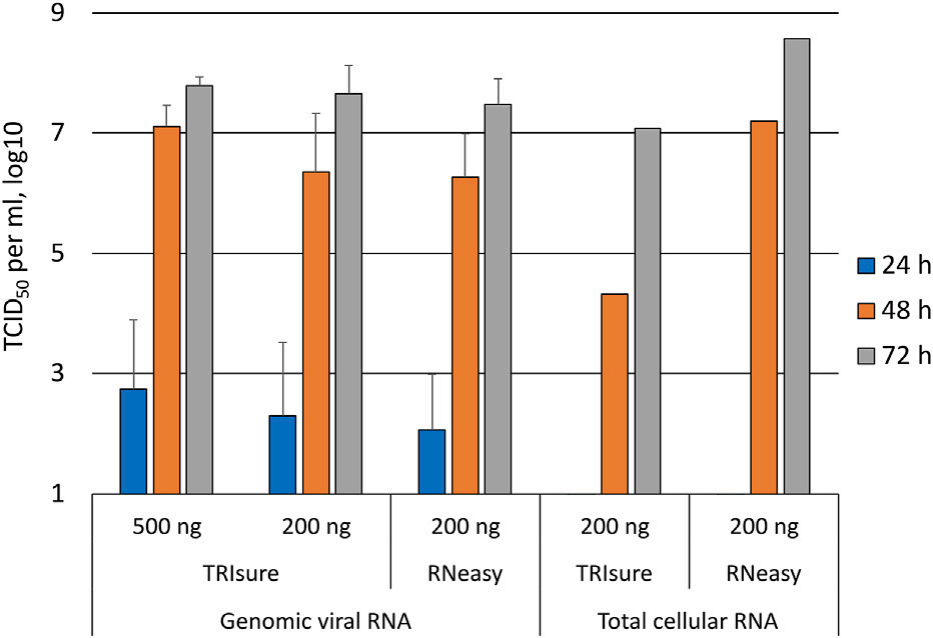
Daily follow-up of the appearance of infectious viruses into media post-transfection. Supernatant samples were collected daily from transfected wells and analyzed with an endpoint dilution assay for the presence of infectious viruses. The figure presents the TCID_50_/ml results from daily samples only from wells in which infectious viruses were observed at 72 h timepoint. The means and standard errors of the means have been calculated from the following number of experiments (the total number of replicates in parenthesis): 500 ng TRIsure, 1 (3); 200 ng TRIsure, 3 (13); 200 ng RNeasy, 4 (14). The results for total cellular RNA transfection are from a single positive well for both TRIsure and RNeasy extracted RNA. TRIsure, RNA extracted with TRIsure reagent; RNeasy, RNA extracted with RNeasy Mini kit.

In a subset of experiments, the transfected cells were monitored daily for the presence of CPE as well as by taking a daily sample from the media that was analyzed by an endpoint dilution assay for virus titers (TCID_50_/ml). After transfecting 200 ng of genomic vRNA per well, infectious viruses were observed in the media 24 h post-transfection in 60%–70% of the wells that showed infectious virus in later timepoints (Figure 3). At 48 h, the TCID_50_ titer was already high, reaching 1 × 10^6^–1 × 10^7^ per ml, and at 72 h post-transfection the TCID_50_ titer was similar to that of the original Fin32 virus stock. In contrast to the TCID_50_ values, CPE was completely absent at the 24 h time point and only very little CPE (5% of the cells in maximum) was observed at 48 h. At 72 h post-transfection the level of CPE varied from 60% to the complete death of all cells.

When the cells were transfected with total cellular RNA from infected cells, wells with an infection were rarely observed. Consequently, the results from daily follow-up samples of transfected cells producing infectious viruses were available only from two individual wells, one for TRIsure-extracted total cellular RNA and one for RNeasy Mini kit-extracted total cellular RNA. In both cases no infectious virus was detected at 24 h, yet the 72 h TCID_50_/ml titer reached similar levels as with genomic vRNA transfections (Figure 3).

## Discussion

The positive-stranded genomic vRNA of coronaviruses has been known to be infectious since the 1970s, and overall, experiments with SARS-CoV-2 in which infectious viruses are or may be propagated are carried out in higher containment level laboratories. However, SARS-CoV-2 RNA has been routinely handled in BSL-2 laboratories world-wide since January 2020, raising the question of the true nature of RNA infectiousness in these samples and sufficient biosafety measures. Our aim was to analyze the ability of samples harboring SARS-CoV-2 genomic RNA to produce infectious viruses when transfected into permissive cells and discuss the biosafety control measures related to these assays.

Our results demonstrate that, in general, transfection with large quantities of genomic vRNA of SARS-CoV-2 is required for the production of infectious viruses. When a large quantity of vRNA containing more than 3 × 10^5^ genomic RNA copies per cell was used, the transfection efficiency for rescuing an infectious virus was up to 100%. Similar results have been reported in studies with *in-vitro*-transcribed vRNA in which the amount of transfected RNA has been high (Thi Nhu Thao et al., 2020). However, we also showed that the mere quantity of vRNA is not the only factor reflecting infectivity. When total cellular RNA extracted from virus-infected cells was transfected, we did observe replicating, infectious viruses, although there was markedly lower amounts of genomic vRNA in samples with total cellular RNA. In studies using *in-vitro*-transcribed vRNA, co-transfection of mRNA coding for coronavirus N protein has been shown to enhance the infectivity of the genomic vRNA (Casais et al., 2001; Yount et al., 2002; 2003; Thi Nhu Thao et al., 2020). During SARS-CoV-2 infection, up to 50% of produced viral subgenomic RNAs may be N gene-specific (Thorne et al., 2022). In our experiments, the presence of N transcripts within the total cellular RNA sample may have been one factor aiding the production of infectious viruses when the genomic vRNA quantity in the sample was lower.

Recently, Haddock and coworkers demonstrated that the kit-extracted SARS-CoV-2 RNA (with AVL buffer, Qiagen) can be infectious already in small quantities (Haddock et al., 2021). We did not observe infectious viruses from transfections with 50 ng per well or less of genomic vRNA extracted with TRIsure. However, when 200 ng of vRNA per well was used, RNeasy Mini kit-extracted and TRIsure-extracted vRNA functioned equally well in producing infectious viruses. These results demonstrate further that different transfection conditions and possibly also RNA extraction methods can affect the results considerably.

We also investigated the timeline of infectious virus production after transfection. Already at 24 h post-transfection there were infectious viruses present in the media, and on the next day the virus titer was already very high. In contrast, no CPE was visible at 24 h, and at 48 h, CPE was very modest. Similarly, Yount and coworkers showed that when *in-vitro* transcribed SARS-CoV RNA was electroporated into cells, infectious viruses were present in the media at 24 h post-transfection (Yount et al., 2003). The level of CPE at different time points was not recorded, however, at 48 h SARS-CoV-infected cells were detected by immunofluorescence microscopy. In other studies, when large amounts of *in-vitro* transcribed coronavirus RNA was used in transfections, CPE was observed at 48 h to 6 days post-transfection (Thiel et al., 2001; Scobey et al., 2013; Thi Nhu Thao et al., 2020; Xie et al., 2020). Unfortunately, in these studies the daily virus growth was not analyzed. Collectively, the data shows that the replication of infectious viruses may start rapidly after transfection, but a visually detectable cytopathic effect follows later. Therefore, also the transfection experiments with a shorter duration, as in studies for innate immune responses, should be carefully assessed whether to be categorized as a propagative work.

The efficiency of the genomic vRNA from other SARS-CoV-2 variants to produce infectious viruses when transfected was not studied here. The beta variant Fin32 replicates efficiently in our cell culture system although it lacks the mutations R203K + G204R in N protein that has been recently shown to enhance virus replication (Johnson et al., 2022). Any conclusions for the possible replication enhancement in transfections based on sequence differences between variants cannot be extrapolated from our results. On the other hand, the possibility of even more efficient production of infectious viruses needs to be kept in mind when biosafety of transfections is evaluated for other variants.

Plain RNA without the protection from a transfection reagent was rapidly degraded in the cell culture. Our RT-qPCR data demonstrated that when naked vRNA was added to cell culture wells at a concentration corresponding to >1 × 10^10^ copies of SARS-CoV-2 genomes, no vRNA was detected after 1 hour by RT-qPCR. This over 9-log reduction in 1 hour shows that unprotected vRNA is very vulnerable to RNases, and it is highly unlikely that plain vRNA would be able to get into the cells and initiate the production of infectious viruses. In addition, total cellular RNA was as easily degraded as the pure genomic vRNA.

The current WHO guidelines states that SARS-CoV-2 RNA can be handled at BSL-2, although propagative work should be done in BSL-3 (World Health Organization, 2021). Nevertheless, in the biosafety perspective it is critical to consider how the RNA is used in downstream applications. For example, RT-qPCR and transfection studies have very different outcome regarding the presence of infectious viruses. Our data shows that when transfecting large amounts of genomic vRNA, the production of infectious viruses is very efficient. Similar large quantities of vRNA are used in the studies with infectious cDNA clones, and overall, such studies should be carried out in BSL-3 facilities. On the other hand, we showed that the vRNA quantity alone is not the only factor in the process, and even a small amount of genomic vRNA may be sufficient for virus replication, especially when the RNA is derived from infected cells. Furthermore, virus replication can start rapidly after transfection, and there can be plenty of infectious viruses in the cell culture media without any apparent CPE in the cells. In these situations, transfections with less vRNA or experiments with a shorter timeline, the biosafety aspects should not be neglected either. Taken together, it is important that all transfection experiments with samples containing genomic SARS-CoV-2 RNA are categorized as propagative work and that the work is conducted only in a higher containment level laboratory to minimize the biosafety risks.

## Data Availability

The datasets presented in this study can be found in online repositories. The names of the repository/repositories and accession number(s) can be found in the article.
